# Prevalence of iron-deficiency anemia in pregnant women with various thalassemia genotypes: Thoughts on iron supplementation in pregnant women with thalassemia genes

**DOI:** 10.3389/fnut.2022.1005951

**Published:** 2022-11-17

**Authors:** Min Wang, Xiaozhuang Zhang, Yanhong Zhao, Yubin Zhang, Yan Lin, Meifang Xiao, Ling Li

**Affiliations:** Hainan Women and Children’s Medical Center, Haikou, China

**Keywords:** iron-deficiency anemia (IDA), pregnancy, prevalence, iron supplementation, thalassemia

## Abstract

**Background:**

There are limited studies on iron-deficiency anemia (IDA) in carriers of various thalassemia genotypes. However, for pregnant women (PW) with high iron demand, ignoring the phenomenon of carrying the thalassemia genes combined with IDA may lead to adverse pregnancy outcomes.

**Methods:**

The hematological phenotype indexes of 15,051 PW who received a prenatal diagnosis of thalassemia in our hospital were analyzed, and the plasma ferritin (PF) of 714 anemic pregnant women (APW) was determined.

**Results:**

The results showed that 87.43% of APW without thalassemia suffered from IDA. Among APW with various thalassemia genotypes, we found that 40.00∼77.78% of subjects with α-thalassemia silent genotypes [α^CS (or QS)^α/αα (40.00%), –α^3.7(or^ ^4.2)^/αα (57.65%), and α^WS^α/αα (77.78%)] and 18.18∼84.21% of subjects with α-thalassemia minor genotypes [α^CS (or QS)^α/–α^3.7(or^ ^4.2)^ (18.18%), –α^3.7(or^ ^4.2)^/–α^3.7(or^ ^4.2)^ (40.00%), αα/–^SEA^ (44.55%), and α^WS^α/–α^3.7(or^ ^4.2)^ (84.21%)] developed IDA, while in subjects with α-thalassemia intermedia genotypes, only α^WS^α/–^SEA^ was associated with IDA, with an incidence of 16.67%. However, the incidence of IDA in APW with common β-thalassemia minor genotypes (β^CD17(A>T)^/β, β^CD41/42 (–TTCT)^/β, β^CD71/72(+*A*)^/β, β^IVS–*II*–654(C>T)^/β, and β^–28(A>G)^/β) was less than 10.85%. In addition, the APW with β-thalassemia minor had a higher PF level than the APW without thalassemia.

**Conclusion:**

Our study is the first to reveal differences in the prevalence of IDA among PW with various thalassemia genotypes, indicating that the possibility of IDA should be fully considered when managing PW with α-thalassemia silent or minor genotypes in high-risk areas, and that iron supplementation should be monitored dynamically for PW with β-thalassemia minor genotypes.

## Introduction

Thalassemia is one of the most widespread autosomal monogenic diseases in the world. In clinical practice, α-thalassemia and β-thalassemia are the most common subtypes, and the hematological phenotype of different genotypes showed obvious heterogeneity (1). At the molecular level, depending on the number of functional α- and β-globin genes retained, α-gene defects can be classified into α^+^ and α^0^, and β-gene defects can be classified into β^+^ and β^0^ (2, 3). Carriers of α-thalassemia silent genotypes (α^+^α/αα) often have no clinical phenotype and are mostly found in population screening. Carriers of α- or β-thalassemia minor genotypes (α^+^α/α^+^α, α^0^α/αα, β^+^/β, and β^0^/β) present with microcytosis, mild anemia or no anemia, and no treatment is required. Patients with α- or β-thalassemia intermedia genotypes (α^0^α/α^+^α, β^+^/β^+^, and some β^0^/β^+^) can develop mild to moderate anemia with age and some require lifelong transfusion therapy. While infants with α-thalassemia major (α^0^/α^0^) are usually stillborn or die quickly after birth, and infants with β-thalassemia major genotypes (some β^0^/β^+^, β^0^/β^0^) develop progressive hemolytic anemia 3–6 months after birth and die before 5 years if untreated.

Iron-deficiency anemia (IDA) is caused by insufficient iron intake, poor iron absorption, or excessive iron loss. Because of the increasing demand for iron in women’s own and fetal development during pregnancy, the WHO recommends maternal iron supplements to avoid adverse pregnancy outcomes caused by IDA (4–6). However, due to the combined effects of chronic hemolysis, iron utilization disorder, or repeated blood transfusion in thalassemia carriers, iron supplementation is generally not recommended since it may lead to different levels of iron overload. In any case, for a particular population with high iron demand, such as children and pregnant women (PW), ignoring the phenomenon of carrying thalassemia genes combined with IDA may lead to adverse clinical outcomes. In addition, we searched relevant literature and found that there was no study to report differences in IDA prevalence among PW with various genotypes of thalassemia. Therefore, this paper aims to reveal the difference in iron metabolism of PW with various thalassemia genotypes and provide scientific reference for clinical individualized management of such a population.

## Materials and methods

### Participants

This study was conducted based on the “Hainan Pregnancy Thalassemia Screening Program,” which was implemented in 2019 in Hainan Province to provide free blood routine tests and genetic diagnosis of thalassemia for PW and their partners. All participants were recruited from medical institutions within 14 cities and counties in Hainan. Cases with MCV values less than 82 fL were considered possible carriers of thalassemia (7). The subjects of this study were PW who underwent prenatal genetic diagnosis of thalassemia in Hainan Women and Children’s Medical Center from August 2019 to December 2021. The age of the PW ranged from 13 to 54 years, with a median age of 28 years. The gestational ages of PW were between 4 and 39 weeks, and early pregnancy (4∼12 weeks) accounted for about 53.9%, the second trimester (13∼27 weeks) accounted for about 40.8%, the third trimester (after 28 weeks) accounted for about 5.3%. The majority of subjects were screened for hematological parameters and thalassemia genes for the first time during pregnancy, so most subjects did not receive professional iron supplementation before screening, but multivitamin supplementation could not be excluded. All studies were approved by the Ethics Committee of Hainan Women and Children’s Medical Center.

### Measurements of hematological phenotype indexes and plasma ferritin levels, as well as gene diagnosis of thalassemia

According to the recommendation of the World Health Organization, anemia in pregnancy can be diagnosed when the Hb concentration of the PW is less than 110 g/L. Meanwhile, according to the Guidelines for Diagnosis and Treatment of Iron Deficiency (ID) and Iron-Deficiency Anemia during Pregnancy in China (2014), when the plasma ferritin (PF) level is below 30 ng/mL, early iron depletion is considered and iron supplementation should be performed (8). Peripheral blood samples of 4 mL volume were taken from all participants and collected into two Ethylenediamine Tetraacetic Acid (EDTA) anti-coagulated tubes on average. The hematological phenotype indexes were measured and analyzed by the hemocyte analyzer at the medical institutions where the subjects were located. When the MCV value of the pregnant woman or her partner was less than 82 fL, the peripheral blood of both the couple was collected. Then the peripheral blood samples were immediately transported to our center by cold chain (The temperature was 4∼8°C, and the specimen transportation was generally completed within 2∼5 days) for the genetic diagnosis of thalassemia. If the Hb concentration of the pregnant woman was below 110 g/L and sufficient qualified plasma could be successfully isolated, the plasma was collected for ferritin level determination. A flow chart of this study is shown in Supplementary Figure 1. PF levels were measured using the Elecsys Ferritin kit (Roche Diagnostics GmbH, Mannheim, Germany). The gap-polymerase chain reaction (PCR) was used to identify α-globin gene deletion mutations (#20193401915, Yaneng Biosciences, Shenzhen, China). Reverse dot-blot hybridization was used to identify non-deletional mutations of α- and β-thalassemia (#20173401107, #20163400463, Yaneng Biosciences, Shenzhen, China). A deoxyribonucleic acid (DNA) sequence was used to detect rare thalassemia gene mutations.

### Statistics

Graphpad Prism 8 (GraphPad Software, La Jolla, CA) was used for statistical analyses. Comparison of composition ratios between two or more groups using Fisher’s exact test or the chi-square test followed by the Bonferroni method. The unpaired Student’s *t*-tests or Welch’s *t*-tests were used to determine statistical significance between two groups. If the variance of multiple groups was the same, a one-way ANOVA was used to compare the mean of multiple groups; otherwise, the Kruskal–Wallis *H*-test was used. The data were given as Mean ± SD. A *P*-value of < 0.05 was considered significant.

## Results

### Analysis of anemia prevalence and hematological phenotype indexes in pregnant women with various thalassemia genotypes

**FIGURE 1:**
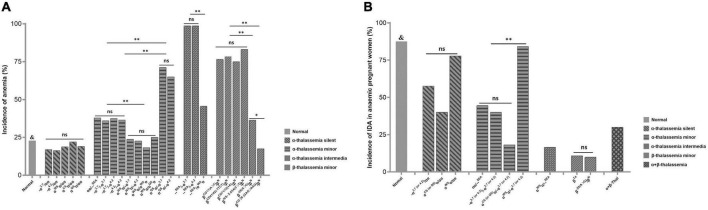
**(A)** The prevalence of anemia among PW with various genotypes of thalassemia ^&^*P* < 0.05 vs. groups of –α^3.7^/αα, –α^4.2^/αα, αα/–^SEA^, –α^3.7^/–α^3.7^, –α^3.7^/–α^4.2^, –α^4.2^/–α^4.2^, α^QS^α/–^3.7^, α^QS^α/–^4.2^, –α^3.7^/–^SEA^, –α^4.2^/–^SEA^, α^WS^α/–^SEA^, β^IVS– II– 654(C>T)^/β, β^CD41/42 (– TTCT)^/β, β^CD17(A>T)^/β, β^CD71/72(+A)^/β, and β^– 28(A>G)^/β. ns, no significant; PW, pregnant women. *P < 0.05; ^**^P < 0.01. **(B)** The incidence of IDA in APW with various genotypes of thalassemia. ^&^*P* < 0.05 vs. groups of –α^(3.7^ ^or^ ^4.2)^/αα, α^(CS^ ^or^ ^QS)^α/αα, αα/–^SEA^, –α^(3.7^ ^or^ ^4.2)^/–α^(3.7^ ^or^ ^4.2)^, α^(CS^ ^or^ ^QS)^α/–α^(3.7^ ^or^ ^4.2)^, α^WS^α/–^SEA^, β^T4^/β, β-28(A>G)/β, and α + β-thalassemia. **-α** ^3.7 (or^ ^4.2)^**/αα** : –α^3.7^/αα and –α^4.2^/αα were combined into one group. α ^CS (or^ ^QS)^α /αα : α^CS^α/αα and α^QS^α/αα were combined into one group. **-α ^3.7 (or^ ^4.2)^/-α ^3.7 (or^ ^4.2)^**: –α^3.7^/–α^3.7^, –α^3.7^/–α^4.2^, and –α^4.2^/–α^4.2^ were combined into one group. **α ^CS (or^ ^QS)^α /-α ^3.7 (or^ ^4.2)^**: α^CS^α/–α^3.7^, α^CS^α/–α^4.2^, α^QS^α/–α^3.7^, and α^QS^α/–α^4.2^ were combined into one group. **α ^WS^α /-α ^3.7 (or^ ^4.2)^**: α^WS^α/–α^3.7^, and α^WS^α/–α^4.2^ were combined into one group. **-α ^3.7 (or^ ^4.2)^/–^SEA^**: –α^3.7^/–^SEA^ and –α^4.2^/–^SEA^ were combined into one group. **β ^T4^**: β^IVS– *II*– 654(C>T)^/β, β^CD41/42 (– TTCT)^/β, β^CD17(A>T)^/β, and β^CD71/72(+A)^/β were combined into one group. ns, no significant; IDA, iron-deficiency anemia; APW, anemic pregnant women. *P < 0.05; ^**^P < 0.01.

The anemia rate and hematological phenotype indexes of 15,051 PW who underwent prenatal diagnosis of thalassemia in our hospital from August 2019 to December 2021 were analyzed. The results showed that a total of 1,219 anemic pregnant women (APW) were detected in 5,365 PW without thalassemia genotypes, and the prevalence of anemia in PW without thalassemia was 22.72%. The anemia rate in PW with five common α-thalassemia silent genotypes ranged from 16.25 to 22.03%, and no statistical difference was found. For α-thalassemia minor genotypes, the anemia rates of α^WS^α/α*^QS^*α, α*^WS^*α/α^WS^α, α*^WS^*α/–α^3.7^, and α*^WS^*α/–α^4.2^ were significantly lower than those of other α-thalassemia minor genotypes, ranging from 18.18 to 25.00%. The anemia rates of αα/–*^SEA^*, –α^3.7^/–α^3.7^, –α^3.7^/–α^4.2^, and –α^4.2^/–α^4.2^ ranged from 35.96 to 37.82% (*P* > 0.05). While α^QS^α/–α^3.7^ (71.19%) and α*^QS^*α/–α^4.2^ (65.00%) had higher anemia rates than other α-thalassemia minor genotypes. For α-thalassemia intermedia genotypes, the anemia rates of –α^3.7^/–*^SEA^*, –α^4.2^/–*^SEA^*, and α^WS^α/–*^SEA^* were 98.65, 98.65, and 45.61%, respectively, and α^WS^α/–*^SEA^* had a significantly lower anemia rate than the former two genotypes. Among six common β-thalassemia minor genotypes, the incidence of anemia from high to low was β*^IVS^*^–II–654(C>T)^/β (83.04%), β^CD41/42 (–*TTCT*)^/β (78.44%), β^CD17(A>T)^/β (76.54%), β^CD71/72(+A)^/β (75.00%), β^–28(A>G)^/β (36.29%), and β^CD26 (GAG>*AAG*)^/β (17.50%), and the latter two genotypes had significantly lower anemia rates than the first four. The anemia rates of PW with β-thalassemia minor genotypes were significantly higher than that of PW without thalassemia, except β^CD26 (GAG>AAG)^/β. The anemia prevalence among PW with various genotypes of thalassemia was shown in Figure 1A.

Analysis of hematological phenotype indexes showed that α^WS^α caused the least severe hematology phenotype among five common α^+^-thalassemia genes. When α^WS^α was co-inherited with other α^+^-thalassemia genes, the Hb and MCH levels of the genotypes (α^WS^α/–α^3.7^, α^WS^α/–α^4.2^, α^WS^α/α^WS^α, and α^WS^α/α^QS^α) were higher than those of other common α-thalassemia minor genotypes (αα/–^SEA^, –α^3.7^/–α^3.7^, –α^4.2^/–α^4.2^, –α^3.7^/–α^4.2^, α^QS^α/–α^3.7^, α^QS^α/–α^4.2^). When α^WS^α was co-inherited with –^SEA^, the Hb and MCH levels of the genotype (α^WS^α/–^SEA^) were significantly higher than those of –^SEA^/–α^3.7^ and –^SEA^/–α^4.2^. The severity of hematological phenotypes caused by –α^3.7^ and –α^4.2^ tend to be consistent. There was no significant difference in Hb and MCH levels of –α^3.7^/–α^3.7^, –α^4.2^/–α^4.2^, –α^3.7^/–α^4.2^, and the values of hematological phenotype indexes caused by those three phenotypes were similar to that of αα/–^SEA^. α*^CS^*α and α^QS^α caused the most severe hematologic phenotype among five common α^+^ genes. When α^QS^α was co-inherited with –α^3.7^ or –α^4.2^, the Hb and MCH levels of the genotypes (α^QS^α/–α^3.7^, α^QS^α/–α^4.2^) were significantly lower than those of other common α-thalassemia minor genotypes. For common β-thalassemia minor genotypes, there was no significant difference in Hb and MCH levels among β^CD17(A>T)^/β, β^CD41/42 (–*TTCT*)^/β, β^CD71/72(+A)^/β, and β^IVS–II–654(C>T)^/β, but all were obviously lower than that of PW without thalassemia. The Hb and MCH levels of β^–28(A>G)^/β and β^CD26 (GAG>AAG)^/β were significantly higher than those of the above four β-thalassemia genotypes. The analysis of hematological phenotype indexes among PW with various genotypes of thalassemia is shown in Table 1.

**TABLE 1:** The value of Hb and MCH in PW with or without thalassemia genotypes.

Genotype	Cases (*n*)	RBC (×10^12^/L)	Hb (g/L)	MCV (fL)	MCH (g/L)
Normal	5,365	4.36 ± 0.44	119.4 ± 15.2^a^	82.51 ± 7.42	27.51 ± 3.26^b^
–α^3.7^/αα	1,757	4.58 ± 0.44	119.6 ± 12.4	79.28 ± 3.94	26.16 ± 1.85
–α^4.2^/αα	1,674	4.61 ± 0.45	119.9 ± 11.9	79.25 ± 4.04	26.15 ± 1.94
α^WS^α/αα	698	4.54 ± 0.49	119.5 ± 12.8	79.77 ± 5.29	26.49 ± 2.42^d^
α^CS^α/αα	59	4.48 ± 0.32	116.3 ± 8.8	79.34 ± 3.24	25.94 ± 1.47
α^QS^α/αα	299	4.85 ± 0.42	117.6 ± 10.3^c^	74.81 ± 3.77	24.37 ± 1.66^e^
–^SEA^/αα	1,716	5.20 ± 0.5	111.8 ± 9.8^f^	67.54 ± 3.75	21.58 ± 1.47^h^
–α^3.7^/–α^3.7^	292	4.90 ± 0.46	112.1 ± 10.5^f^	71.49 ± 4.0	22.96 ± 1.61^i^
–α^3.7^/–α^4.2^	526	4.92 ± 0.50	112.1 ± 10.2^f^	71.10 ± 3.72	22.85 ± 1.43^i^
–α^4.2^/–α^4.2^	266	4.98 ± 0.51	112.6 ± 11.4^f^	70.93 ± 3.78	22.82 ± 1.85^i^
α^WS^α/–α^3.7^	325	4.64 ± 0.47	117.2 ± 11.7^g^	77.74 ± 3.92	25.41 ± 1.88
α^WS^α/–α^4.2^	283	4.67 ± 0.43	117.3 ± 10.6^g^	77.32 ± 4.36	25.15 ± 1.72
α^WS^α/α^WS^α	88	4.61 ± 0.49	119.8 ± 14.3^g^	79.14 ± 4.41	26.21 ± 2.16
α^WS^α/α^QS^α	32	4.99 ± 0.47	117.1 ± 10.7^g^	72.50 ± 2.99	23.47 ± 0.82^j^
α^QS^α/–α^3.7^	59	5.06 ± 0.58	105.1 ± 9.8	65.55 ± 3.93	21.08 ± 1.89^k^
α^QS^α/–α^4.2^	40	5.21 ± 0.55	105.5 ± 10.9	64.50 ± 3.88	20.59 ± 2.51^k^
–^SEA^/–α^3.7^	74	4.74 ± 0.60	85.53 ± 9.6	60.00 ± 6.40	18.94 ± 3.42
–^SEA^/–α^4.2^	74	4.73 ± 0.49	87.05 ± 9.8	61.33 ± 6.41	18.75 ± 2.43
–^SEA^/α^WS^α	57	5.14 ± 0.50	108.1 ± 10.7^l^	65.95 ± 4.54	21.06 ± 1.59^l^
β^CD17 (A^^>^^T)^/β	81	5.13 ± 0.58	103.2 ± 8.9	63.28 ± 4.04	20.31 ± 1.53
β^CD41/42 (–TTCT)^/β	807	5.06 ± 0.60	102.1 ± 10.0	63.32 ± 3.77	20.36 ± 1.61
β^CD71/72 (+A)^/β	68	5.03 ± 0.58	102.2 ± 10.6	63.52 ± 3.14	20.34 ± 1.19
β^IVS–*II*–654 (C^^>^^T)^/β	112	5.02 ± 0.48	102.1 ± 7.9	63.46 ± 3.29	20.46 ± 1.33
β^–28 (A^^>^^G)^/β	259	4.93 ± 0.44	112.6 ± 9.3^m^	70.69 ± 3.46	22.92 ± 1.26^m^
β^CD^ ^26 (GAG^^>^^AAG)^/β	40	4.63 ± 0.35	117.1 ± 8.3^m^	76.08 ± 3.19	25.49 ± 1.52^m^

The value of Hb and MCH were compared among different groups. **(1)** ^a^The Hb level of Normal group was higher than those of α^QS^α/αα, –^SEA^/αα, –α^3.7^/–α^3.7^, –α^3.7^/–α^4.2^, –α^4.2^/–α^4.2^, α^WS^α/–α^3.7^, α^WS^α/–α^4.2^, α^QS^α/–α^3.7^, α^QS^α/–α^4.2^, –^SEA^/α^WS^α, –^SEA^/–α^3.7^, –^SEA^/–α^4.2^, β^CD17 (A>T)^/β, β^CD41/42 (–TTCT)^/β, β^CD71/72 (+A)^/β, β^IVS–II–654 (C>T)^/β, and β^–28 (A>G)^/β groups (P < 0.05); ^b^the MCH level of Normal group was higher than those of all other groups (P < 0.05). **(2)** Among α-thalassemia silent genotypes, ^c^the Hb level of α^QS^α/αα was lower than those of –α^3.7^/αα, –α^4.2^/αα, and α^WS^α/αα (P < 0.05); ^d^the MCH level of α^WS^α/αα was higher than those of –α^3.7^/αα, –α^4.2^/αα, α^QS^α/αα, and α^CS^α/αα (P < 0.05); ^e^the MCH level of α^QS^α/αα was lower than those of –α^3.7^/αα, –α^4.2^/αα, and α^CS^α/αα (P < 0.05). **(3)** Among α-thalassemia minor genotypes, ^f^the Hb levels of –^SEA^/αα, –α^3.7^/–α^3.7^, –α^3.7^/–α^4.2^, and –α^4.2^/–α^4.2^ were lower than those of α^WS^α/–α^3.7^, α^WS^α/–α^4.2^, and α^WS^α/α^WS^α, while was higher than those of α^QS^α/–α^3.7^ groups (P < 0.05); ^g^the Hb levels of α^WS^α/–α^3.7^, α^WS^α/–α^4.2^, α^WS^α/α^WS^α, and α^WS^α/α^QS^α were higher than those of α^QS^α/–α^3.7^, α^QS^α/–α^4.2^ (P < 0.05); ^h^the MCH level of –^SEA^/αα was higher than that of α^QS^α/–α^4.2^, while was lower than those of –α^3.7^/–α^3.7^, –α^3.7^/–α^4.2^, –α^4.2^/–α^4.2^, α^WS^α/–α^3.7^, α^WS^α/–α^4.2^, α^WS^α/α^WS^α, and α^WS^α/α^QS^α (P < 0.05); ^i^the MCH levels of –α^3.7^/–α^3.7^, –α^3.7^/–α^4.2^, and –α^4.2^/–α^4.2^ were lower than those of α^WS^α/–α^3.7^, α^WS^α/–α^4.2^, and α^WS^α/α^WS^α, while were higher than those of α^QS^α/–α^3.7^, α^QS^α/–α^4.2^ (P < 0.05); ^j^the MCH level of α^WS^α/α^QS^α was lower than that of α^WS^α/α^WS^α (P < 0.05); ^k^the MCH levels of α^QS^α/–α^3.7^, α^QS^α/–α^4.2^ were lower than those of α^WS^α/–α^3.7^, α^WS^α/–α^4.2^, α^WS^α/α^WS^α, and α^WS^α/α^QS^α (P < 0.05). **(4)** Among the α-thalassemia intermediate genotypes, ^l^the Hb and MCH levels of –^SEA^/α^WS^α were lower than those of –^SEA^/–α^3.7^, and –^SEA^/–α^4.2^ (P < 0.05). **(5)** Among the β-thalassemia minor genotypes, ^m^the Hb and MCH levels of β^–28 (A>G)^/β, β^CD^ ^26 (GAG>AAG)^/β were higher than those of β^CD17 (A>T)^/β, β^CD41/42 (–TTCT)^/β, β^CD71/72 (+A)^/β, and β^IVS–II–654 (C>T)^/β (P < 0.05). n, number; PW, pregnant women; RBC, red blood cell; Hb, hemoglobin; MCV, mean corpuscular volume; MCH, mean corpuscular hemoglobin concentration.

### Incidence of iron deficiency in anemic pregnant women with various genotypes of thalassemia

PF levels were measured in 714 APW who underwent a prenatal diagnosis of thalassemia in our hospital. Some genotypes were combined and grouped according to similar anemia prevalence and hematological parameters as shown in Table 2. The results showed that 87.43% of anemia in APW without thalassemia was caused by ID. There was no significant difference in the incidence of ID among the five α-thalassemia silent genotypes, but it can be seen that the ID rate was the highest in α^WS^α/αα (77.78%), followed by –α^(3.7 or^ ^4.2)^/αα (57.65%), and α^CS (or QS)^α/αα (40.00%). For α-thalassemia minor genotypes, the ID rate of α^WS^α/–α^(3.7 or^ ^4.2)^ (84.21%) was significantly higher than those of other α-thalassemia minor genotypes. Although the incidence of ID was not significant among carriers of –^SEA^/αα(44.55%), –α^(3.7 or^ ^4.2)^/–α^(3.7 or^ ^4.2)^ (40.00%), and α*^CS (or QS)^*α/–α^(3.7 or^ ^4.2)^ (18.18%), it is seen that the ID rate of α*^CS (or QS)^*α/–α^(3.7 or^ ^4.2)^ was obviously lower than those of the other above genotypes. In addition, two APW with α^WS^α/α^WS^α and one APW with α^QS^α/α^QS^α were not included in statistical analysis due to the small number of cases. Two APW with α^WS^α/α^WS^α developed IDA, while APW with α^QS^α/α^QS^α had mild anemia and without ID. For α-thalassemia intermediate genotypes, no ID was found in carriers of –α^3.7^/–^SEA^, –α^4.2^/–^SEA^, and α*^CS^*α/–^SEA^, while ID was found in one of six carriers of α^WS^α/–^SEA^. For common β-thalassemia minor genotypes, β^IVS–II–654(C>T)^/β, β^CD41/42 (–TTCT)^/β, β^CD17(A>T)^/β, and β^CD71/72(+*A*)^/β were combined into one group due to similar anemia prevalence and Hb, MCH levels. The ID rates of the above four genotypes and β^–28(A>G)^/β were 10.85 and 10.00%, respectively. One APW with β^CD26 (GAG>*AAG*)^/β was not included in the statistical analysis, and the carrier did not develop ID. The incidence of ID in APW with α + β-thalassemia genotypes included in this study was 30%. The results are shown in Figure 1B. In addition, by comparing Hb levels of APW with or without ID, we found that in general, the Hb levels of APW with ID were slightly lower than those of APW without ID. For APW with thalassemia minor genotypes, the carriers of α^CS (or^ ^QS)^α/–α^3.7 (or^ ^4.2)^ and common β-thalassemia minor genotypes had obviously higher PF levels than APW without thalassemia (Table 2).

**TABLE 2:** The levels of Hb and PF in APW with or without ID.

	Hb^PF^^<^^30^ (g/L)		Hb^PF^^≥^^30^ (g/L)				PF* (ng/mL)	PF (ng/mL)
						
Genotype	Mean ± SD	*n*	Mean ± SD	*n*	*t*	*p*	Mean ± SD	Median, (P_25_; P_75_)
Normal	97.1 ± 11.0	160	102.6 ± 8.7	23	2.294	<0.05	91.91 ± 64.40^a^	11.70, (8.40; 17.60)
–α^3^^.7 (or^ ^4^^.2)^/αα	98.7 ± 9.2	49	105.1 ± 4.2	36	4.275	<0.01	82.09 ± 46.62^a^	18.20, (8.71; 57.80)^a^, ^c^
α^CS (or^ ^QS)^α/αα	/	2	/	3	/	/	/	32.00, (9.37; 45.30)^a^
α^WS^α/αα	95.1 ± 14.6	14	/	4	/	/	/	13.25, (8.43, 28.33)^a^, ^c^
αα/–^SEA^	99.6 ± 7.6	45	103.7 ± 4.8	56	3.181	<0.01	96.82 ± 55.65^a^	34.10, (17.10; 90.60)^a^, ^b^, ^d^
–α^3^^.7 (or^ ^4^^.2)^/–α^3^^.7 (or^ ^4^^.2)^	98.5 ± 9.1	16	101.6 ± 7.4	24	1.196	>0.05	99.26 ± 81.13^a^	38.40, (15.40; 87.40)^a^, ^b^
α^CS (or^ ^QS)^α/–α^3^^.7 (or^ ^4^^.2)^	/	2	100.9 ± 4.9	9	/	/	135.90 ± 82.67	79.10, (30.80; 237.00)^b^
α^WS^α/–α^3^^.7 (or^ ^4^^.2)^	99.8 ± 8.2	16	/	3	/	/	/	13.80, (9.98; 20.90)^a^, ^c^
α^WS^α/α^WS^α	/	2	/	0	/	/	/	/
α^QS^α/α^QS^α	/	0	/	1	/	/	/	/
–α^3^^.7 (or^ ^4^^.2)^/–^SEA^	/	0	88.27 ± 7.2	22	/	/	283.2 ± 131.3	270.90, (186.30; 353.00)^b^
α^CS^α/–^SEA^	/	0	/	1	/	/	/	/
α^WS^α/–^SEA^	/	1	99.80 ± 5.4	5	/	/	187.6 ± 129.6	95.65, (70.73; 317.80)^b^
β^T4^/β	94.9 ± 8.1	14	99.50 ± 6.9	115	2.325	<0.05	122.9 ± 72.71^a^	95.45, (53.05; 140.90)^b^
β^–28(A>G)^/β	/	2	103.70 ± 4.1	18	/	/	119.00 ± 75.21^a^	80.15, (52.00; 188.50)^b^
β^CD26 (GAG>AAG)^/β	/	0	/	1	/	/	/	/

Hb^PF<30^: The Hb levels in APW with ID (PF < 30 ng/mL). Hb^PF≥30^: the Hb levels in APW without ID (PF ≥ 30 ng/mL). PF*: the PF levels in APW without ID (PF ≥ 30 ng/mL). PF: the PF levels in all APW. –α^3.7 (or 4.2)^/αα: –α^3.7^/αα and –α^4.2^/αα were combined into one group. α^CS (or QS)^α/αα: α^CS^α/αα and α^QS^α/αα were combined into one group. –α^3.7 (or 4.2)^/–α^3.7 (or 4.2)^: –α^3.7^/–α^3.7^, –α^3.7^/–α^4.2^, and –α^4.2^/–α^4.2^ were combined into one group. α^CS (or QS)^α/–α^3.7 (or 4.2)^: α^CS^α/–α^3.7^, α^CS^α/–α^4.2^, α^QS^α/–α^3.7^, and α^QS^α/–α^4.2^ were combined into one group. α^WS^α/–α^3.7 (or 4.2)^: α^WS^α/–α^3.7^, and α^WS^α/–α^4.2^ were combined into one group. –α^3.7 (or 4.2)^/–^SEA^: –α^3.7^/–^SEA^, and –α^4.2^/–^SEA^ were combined into one group. β^T4^/β: β^IVS–II–654(C>T)^/β, β^CD41/42 (–TTCT)^/β, β^CD17(A>T)^/β, and β^CD71/72(+A)^/β were combined into one group. ^a^P < 0.05 vs. the group of –α^3.7 (or 4.2)^/–^SEA^. ^b^P < 0.05 vs. the Normal group. ^c^P < 0.05 vs. the groups of β^T4^/β and β^–28(A>G)^/β. ^d^P < 0.05 vs. the group of β^T4^/β./: statistical analysis was performed when the sample size was greater than 5. Hb, hemoglobin; PF, plasma ferritin; APW, anemic pregnant women; ID, iron deficiency.

## Discussion

### Analysis of anemia prevalence and hematological phenotype indexes of pregnant women with various thalassemia genotypes

Hainan Island is the only tropical island province in China. Our previous work shows that the prevalence of thalassemia in Hainan is 13.72%, ranking the second in China. The high-frequent genotypes of α-thalassemia in Hainan were –α^3.7^/αα, –α^4.2^/αα, αα/–^SEA^, α^WS^α/αα, –α^3.7^/–α^4.2^, α^WS^α/–α^3.7^, –α^3.7^/–α^3.7^, –α^4.2^/–α^4.2^, α^WS^α/–α^4.2^, α^QS^α/αα, and the high-frequent genotypes of β-thalassemia were β^CD41/42 (–TTCT)^/β, β^–28(A>G)^/β, β^IVS–*II*–654(C>T)^/β, β^CD71/72(+A)^/β, β^CD17(A>T)^/β, β^CD26 (GAG>AAG)^/β in turn (9). Some of the above genotypes are also prevalent worldwide (1, 2). In this study, we analyzed the anemia prevalence and hematological parameters in PW with or without the aforementioned genotypes of thalassemia. The results showed that the anemia rate in PW without thalassemia (22.72%) was much lower than that of PW with thalassemia genotypes, except for the genotypes of α-thalassemia silent and β^CD26 (GAG>AAG)^/β. The anemia prevalence among various genotypes was consistent with the severity of clinical phenotypes caused by different thalassemia genotypes. The more severe the clinical phenotype caused, the higher the anemia rate, and vice versa. Among the five common α^+^-thalassemia genes, α^WS^α caused the least severe hematology phenotype and the lowest anemia rate. When α^WS^α was co-inherited with other α^+^-thalassemia genes, the hematological phenotypes of the genotypes were less severe than those of other common α-thalassemia minor genotypes, and when α^WS^α was co-inherited with –^SEA^, the severity of hematological phenotype caused by the genotype was much less than those of –^SEA^/–α^3.7^ and –^SEA^/–α^4.2^. The hematological phenotypes caused by –α^3.7^ and –α^4.2^ tend to be consistent. The anemia rate among carriers of αα/–^SEA^, –α^3.7^/–α^3.7^, –α^4.2^/–α^4.2^, and –α^3.7^/–α^4.2^ ranged from 35.96 to 37.82% (*P* > 0.05), and no significant differences were observed in Hb levels among those genotypes. α^CS^α and α^QS^α caused the highest anemia rate and the most severe hematologic phenotypes. When α^QS^α was co-inherited with –α^3.7^ or –α^4.2^, the anemia rate and hematological phenotype severity of α^QS^α/–α^3.7^ and α^QS^α/–α^4.2^ were significantly higher than those of other common α-thalassemia minor genotypes and α^WS^α/–^SEA^. The severity of hematological phenotypes caused by different α^+^-thalassemia genes is consistent with the conclusions of previous studies (2). Among β-thalassemia genes, the anemia rate and the Hb and MCH levels caused by β^IVS–*II*–654(C>T)^ (β^+^) were similar to those of common β^0^-thalassemia genes (β^CD41/42 (–TTCT)^, β^CD17(A>T)^, and β^CD71/72(+A)^) (*P* > 0.05). But the anemia rates induced by β^–28(A>G)^ (β^+^) and β^CD26 (GAG>AAG)^ (β^+^) were obviously lower than those of the above four β-genes, and the Hb and MCH levels were significantly higher than those of the above four β-genes. These results also revealed that the anemia prevalence and hematological phenotype severity of the four common β -thalassemia minor genotypes were higher than those of the common α-thalassemia minor genotypes and α^WS^α/–^SEA^ in Hainan Island.

### Prevalence of iron deficiency in anemic pregnant women with various genotypes of thalassemia

In this study, we first analyzed the anemia rate and hematological parameters of PW with different thalassemia genotypes by a large sample study to obtain the general hematological phenotypic characteristics of each genotype. Subsequently, we combined the genotypes with similar hematologic phenotypic characteristics, and further analyzed the differences of ID in APW with various thalassemia genotypes, so as to provide data reference for clinical individualized management of such a population. The PF levels were determined in 714 APW who underwent prenatal diagnosis of thalassemia in our hospital. The results suggested that the incidence of IDA varied among various genotypes. Among the 183 APW without thalassemia, 87.43% were associated with IDA, indicating that ID is the main cause of anemia in PW in Hainan region. Due to the small number of carriers of specific genotypes included in this study, some genotypes were combined and grouped according to similar anemia rates and hematological parameters. For APW with α-thalassemia silent genotypes, there was no significant difference in the incidence of IDA among different genotypes, but it can be seen that the ID rate was the highest in α^WS^α/αα, followed by –α^(3.7 or 4.2)^/αα, and α^CS (or QS)^α/αα. For APW with α-thalassemia minor genotypes, α^WS^α/–α^(3.7 or 4.2)^ had the highest incidence of IDA, followed by –^SEA^/αα, –α^(3.7 or 4.2)^/–α^(3.7 or 4.2)^, and α^CS (or QS)^α/–α^(3.7 or 4.2)^. Among α-thalassemia intermediate genotypes, ID was only found in carriers of α^WS^α/–^SEA^, and with a low incidence. These results showed that APW with α-thalassemia silent or minor genotypes had higher ID rates and that the prevalence of IDA is inversely correlated with the severity of the hematological phenotype induced by thalassemia genotypes. Among six common β-thalassemia minor genotypes with high prevalence in Hainan, the ID rates of β^CD17(A>T)^/β, β^CD41/42 (–TTCT)^/β, β^CD71/72(+A)^/β, β^IVS–*II*–654(C>T)^/β, and β^–28(A>G)^/β was less than 10.85%. In addition, the APW with common β-thalassemia minor genotypes had obviously higher PF levels than APW without thalassemia, which is consistent with Hoorfar’s study that showed β-thalassemia minor may play a role in improving iron status in females and may lead to iron overload in males (10). Physiological anemia during pregnancy may aggravate the anemia symptoms in carriers of thalassemia minor genotypes, thus affecting maternal and fetal health and even causing long-term adverse effects on newborns. Anymore, a few studies have shown that iron supplementation in carriers of thalassemia with IDA during the third trimester can benefit both mother and baby (11). Although there is no large sample, multicenter randomized controlled study that has yet confirmed the need for routine iron supplementation in PW with thalassemia minor genotypes, we suggest that the ferritin levels of APW with β-thalassemia minor genotypes should be monitored dynamically during iron supplementation, since most carriers do not develop ID and have high PF levels. In conclusion, the possibility of IDA should be fully considered in the clinical management of PW with thalassemia genotypes to avoid adverse pregnancy outcomes, especially for those who with α-thalassemia silent and some minor genotypes.

## Data availability statement

The original contributions presented in this study are included in the article/Supplementary material, further inquiries can be directed to the corresponding authors.

## Ethics statement

The studies involving human participants were reviewed and approved by the Ethics Committee of Hainan Women and Children’s Medical Center. Written informed consent to participate in this study was provided by the participants or their legal guardian/next of kin.

## Author contributions

MW wrote the manuscript. MW, XZ, YHZ, YBZ, and YL interpreted the experiments and analyzed the data. All authors read and approved the final manuscript.

## Abbreviations

PW: Pregnant Women
APW: Anemic Pregnant Women
ID: Iron Deficiency
IDA: Iron-deficiency Anemia
RBC: Red Blood Cell
Hb: Hemoglobin
MCV: Mean Corpuscular Volume
MCH: Mean Corpuscular Hemoglobin
PF: Plasma Ferritin
CS: Constant Spring
QS: Quong Sze
WS: Westmead
SEA: Southeast Asian
CD: Codon
IVS: Intervening Sequences
EDTA: Ethylenediamine Tetraacetic Acid
PCR: Polymerase Chain Reaction
DNA: Deoxyribonucleic Acid.
