# Corrigendum: Prevalence of iron-deficiency anemia in pregnant women with various thalassemia genotypes: thoughts on iron supplementation in pregnant women with thalassemia genes

**DOI:** 10.3389/fnut.2023.1254578

**Published:** 2023-08-30

**Authors:** Min Wang, Xiaozhuang Zhang, Yanhong Zhao, Yubin Zhang, Yan Lin, Meifang Xiao, Ling Li

**Affiliations:** Hainan Women and Children's Medical Center, Haikou, China

**Keywords:** iron-deficiency anemia (IDA), pregnancy, prevalence, iron supplementation, thalassemia

In the published article, there was an error in the name and abbreviation of the measure “mean corpuscular hemoglobin concentration” abbreviated to “MCHC”. It should be “mean corpuscular hemoglobin” and abbreviated to “MCH”. Throughout the text of the article, “mean corpuscular hemoglobin concentration” has been corrected to “mean corpuscular hemoglobin” and the abbreviation “MCHC” has been corrected to “MCH”.

In the published article, there was an error in the header, title and legend for Table 1 as published. “MCHC” should be written as “MCH”.

The corrected Table 1 and its legend appear below.

**Table 1:** The value of Hb and MCH in PW with or without thalassemia genotypes.

**Genotype**	**Cases (*n*)**	**RBC ( × 10^12^/L)**	**Hb (g/L)**	**MCV (fL)**	**MCH (pg)**
Normal	5,365	4.36 ± 0.44	119.4 ± 15.2^a^	82.51 ± 7.42	27.51 ± 3.26^b^
–α^3.7^/αα	1,757	4.58 ± 0.44	119.6 ± 12.4	79.28 ± 3.94	26.16 ± 1.85
–α^4.2^/αα	1,674	4.61 ± 0.45	119.9 ± 11.9	79.25 ± 4.04	26.15 ± 1.94
α^WS^α/αα	698	4.54 ± 0.49	119.5 ± 12.8	79.77 ± 5.29	26.49 ± 2.42^d^
α^CS^α/αα	59	4.48 ± 0.32	116.3 ± 8.8	79.34 ± 3.24	25.94 ± 1.47
α^QS^α/αα	299	4.85 ± 0.42	117.6 ± 10.3^c^	74.81 ± 3.77	24.37 ± 1.66^e^
–^SEA^/αα	1,716	5.20 ± 0.5	111.8 ± 9.8^f^	67.54 ± 3.75	21.58 ± 1.47^h^
–α^3.7^/–α^3.7^	292	4.90 ± 0.46	112.1 ± 10.5^f^	71.49 ± 4.0	22.96 ± 1.61^i^
–α^3.7^/–α^4.2^	526	4.92 ± 0.50	112.1 ± 10.2^f^	71.10 ± 3.72	22.85 ± 1.43^i^
–α^4.2^/–α^4.2^	266	4.98 ± 0.51	112.6 ± 11.4^f^	70.93 ± 3.78	22.82 ± 1.85^i^
α^WS^α/–α^3.7^	325	4.64 ± 0.47	117.2 ± 11.7^g^	77.74 ± 3.92	25.41 ± 1.88
α^WS^α/–α^4.2^	283	4.67 ± 0.43	117.3 ± 10.6^g^	77.32 ± 4.36	25.15 ± 1.72
α^WS^α/α^WS^α	88	4.61 ± 0.49	119.8 ± 14.3^g^	79.14 ± 4.41	26.21 ± 2.16
α^WS^α/α^QS^α	32	4.99 ± 0.47	117.1 ± 10.7^g^	72.50 ± 2.99	23.47 ± 0.82^j^
α^QS^α/–α^3.7^	59	5.06 ± 0.58	105.1 ± 9.8	65.55 ± 3.93	21.08 ± 1.89^k^
α^QS^α/–α^4.2^	40	5.21 ± 0.55	105.5 ± 10.9	64.50 ± 3.88	20.59 ± 2.51^k^
–^SEA^/–α^3.7^	74	4.74 ± 0.60	85.53 ± 9.6	60.00 ± 6.40	18.94 ± 3.42
–^SEA^/–α^4.2^	74	4.73 ± 0.49	87.05 ± 9.8	61.33 ± 6.41	18.75 ± 2.43
–^SEA^/α^WS^α	57	5.14 ± 0.50	108.1 ± 10.7^l^	65.95 ± 4.54	21.06 ± 1.59^l^
β^CD17(A>T)^/β	81	5.13 ± 0.58	103.2 ± 8.9	63.28 ± 4.04	20.31 ± 1.53
β^CD41/42(−*TTCT*)^/β	807	5.06 ± 0.60	102.1 ± 10.0	63.32 ± 3.77	20.36 ± 1.61
β^CD71/72(+A)^/β	68	5.03 ± 0.58	102.2 ± 10.6	63.52 ± 3.14	20.34 ± 1.19
β^IVS − II − 654(C>T)^/β	112	5.02 ± 0.48	102.1 ± 7.9	63.46 ± 3.29	20.46 ± 1.33
β^−28(*A*>*G*)^/β	259	4.93 ± 0.44	112.6 ± 9.3^m^	70.69 ± 3.46	22.92 ± 1.26^m^
β^CD26(GAG>AAG)^/β	40	4.63 ± 0.35	117.1 ± 8.3^m^	76.08 ± 3.19	25.49 ± 1.52^m^

The value of Hb and MCH were compared among different groups. (1) ^a^The Hb level of Normal group was higher than those of α^QS^α/αα, –^SEA^/αα, –α^3.7^/–α^3.7^, –α^3.7^/–α^4.2^, –α^4.2^/–α^4.2^, α^WS^α/–α^3.7^, α^WS^α/–α^4.2^, α^QS^α/–α^3.7^, α^QS^α/–α^4.2^, –^SEA^/α^WS^α, –^SEA^/–α^3.7^, –^SEA^/–α^4.2^, β^CD17(A>*T*)^/β, β^CD41/42(−*TTCT*)^/β, β^CD71/72(+A)^/β, β^IVS − II − 654(C>*T*)^/β, and β^−28(*A*>*G*)^/β groups (*P* < 0.05); ^b^the MCH level of Normal group was higher than those of all other groups (*P* < 0.05). (2) Among α-thalassemia silent genotypes, ^c^the Hb level of α^QS^α/αα was lower than those of –α^3.7^/αα, –α^4.2^/αα, α^WS^α/αα (*P* < 0.05); ^d^the MCH level of α^WS^α/αα was higher than those of –α^3.7^/αα, –α^4.2^/αα, α^QS^α/αα and α^CS^α/αα (*P* < 0.05); ^e^the MCH level of α^QS^α/αα was lower than those of –α^3.7^/αα, –α^4.2^/αα, and α^CS^α/αα (*P* < 0.05). (3) Among α-thalassemia minor genotypes, ^f^the Hb levels of –^SEA^/αα, –α^3.7^/–α^3.7^, –α^3.7^/–α^4.2^, –α^4.2^/–α^4.2^ were lower than those of α^WS^α/–α^3.7^, α^WS^α/–α^4.2^, α^WS^α/α^WS^α, while was higher than those of α^QS^α/–α^3.7^ groups (*P* < 0.05); ^g^the Hb levels of α^WS^α/–α^3.7^, α^WS^α/–α^4.2^, α^WS^α/α^WS^α, α^WS^α/α^QS^α were higher than those of α^QS^α/–α^3.7^, α^QS^α/–α^4.2^ (*P* < 0.05); ^h^the MCH level of –^SEA^/αα was higher than that of α^QS^α/–α^4.2^, while was lower than those of –α^3.7^/–α^3.7^, –α^3.7^/–α^4.2^, –α^4.2^/–α^4.2^, α^WS^α/–α^3.7^, α^WS^α/–α^4.2^, α^WS^α/α^WS^α, α^WS^α/α^QS^α (*P* < 0.05); ^i^the MCH levels of –α^3.7^/–α^3.7^, –α^3.7^/–α^4.2^, –α^4.2^/–α^4.2^ were lower than those of α^WS^α/–α^3.7^, α^WS^α/–α^4.2^, α^WS^α/α^WS^α, while were higher than those of α^QS^α/–α^3.7^, α^QS^α/–α^4.2^ (*P* < 0.05); ^j^the MCH level of α^WS^α/α^QS^α was lower than that of α^WS^α/α^WS^α (*P* < 0.05); ^k^the MCH levels of α^QS^α/–α^3.7^, α^QS^α/–α^4.2^ were lower that those of α^WS^α/–α^3.7^, α^WS^α/–α^4.2^, α^WS^α/α^WS^α, α^WS^α/α^QS^α (*P* < 0.05). (4) Among the α-thalassemia intermediate genotypes, ^l^the Hb and MCH levels of –^SEA^/α^WS^α were lower than those of –^SEA^/–α^3.7^, and –^SEA^/–α^4.2^ (*P* < 0.05); (5) Among the β-thalassemia minor genotypes, ^m^the Hb and MCH levels of β^−28(*A*>*G*)^/β, β^CD26(GAG>AAG)^/β were higher than those of β^CD17(A>*T*)^/β, β^CD41/42(−*TTCT*)^/β, β^CD71/72(+A)^/β, β^IVS − II − 654(C>**T)**^/β (*P* < 0.05). ^n^number; PW, pregnant women; RBC, red blood cell; Hb, hemoglobin; MCV, mean corpuscular volume; MCH, mean corpuscular hemoglobin.

In the published article, there was an error in the legend for Table 2, “The levels of Hb and PF in APW with or without ID” as published. “=” should be written as “≥”; “PF: the PF levels in APW” should be written as “PF: the PF levels in all APW”. The corrected Table 2 and its legend appears below.

**Table 2:** The levels of Hb and PF in APW with or without ID.

**Genotype**	**Hb**^**PF < 30**^ **(g/L)**		**Hb**^**PF≥30**^ **(g/L)**				PF^*^(ng/mL)	**PF (ng/mL)**
	**Mean** ±**SD**	***n***	**Mean** ±**SD**	***n***	**t**	**p**	**Mean** ±**SD**	**Median, (P**_25_ **; P**_75_ **)**
Normal	97.1 ± 11.0	160	102.6 ± 8.7	23	2.294	< 0.05	91.91 ± 64.40^a^	11.70, (8.40; 17.60)
–α^3.7(or4.2)^/αα	98.7 ± 9.2	49	105.1 ± 4.2	36	4.275	< 0.01	82.09 ± 46.62^a^	18.20, (8.71; 57.80)^a, c^
α^CS(orQS)^α/αα	/	2	/	3	/	/	/	32.00, (9.37; 45.30)^a^
α^WS^α/αα	95.1 ± 14.6	14	/	4	/	/	/	13.25, (8.43, 28.33)^a, c^
αα/–^SEA^	99.6 ± 7.6	45	103.7 ± 4.8	56	3.181	< 0.01	96.82 ± 55.65^a^	34.10, (17.10; 90.60)^a, b, d^
–α^3.7(or4.2)^/–α^3.7(or4.2)^	98.5 ± 9.1	16	101.6 ± 7.4	24	1.196	> 0.05	99.26 ± 81.13^a^	38.40, (15.40; 87.40)^a, b^
α^CS(orQS)^α/–α^3.7(or4.2)^	/	2	100.9 ± 4.9	9	/	/	135.90 ± 82.67	79.10, (30.80; 237.00)^b^
α^WS^α/–α^3.7(or4.2)^	99.8 ± 8.2	16	/	3	/	/	/	13.80, (9.98; 20.90)^a, c^
α^WS^α/α^WS^α	/	2	/	0	/	/	/	/
α^QS^α/α^QS^α	/	0	/	1	/	/	/	/
–α^3.7(or4.2)^/–^SEA^	/	0	88.27 ± 7.2	22	/	/	283.2 ± 131.3	270.90, (186.30; 353.00)^b^
α^CS^α/–^SEA^	/	0	/	1	/	/	/	/
α^WS^α/–^SEA^	/	1	99.80 ± 5.4	5	/	/	187.6 ± 129.6	95.65, (70.73; 317.80)^b^
β^T4^/β	94.9 ± 8.1	14	99.50 ± 6.9	115	2.325	< 0.05	122.9 ± 72.71^a^	95.45, (53.05; 140.90)^b^
β^−28(*A*>*G*)^/β	/	2	103.70 ± 4.1	18	/	/	119.00 ± 75.21^a^	80.15, (52.00; 188.50)^b^
β^CD26(GAG>AAG)^/β	/	0	/	1	/	/	/	/

Hb^PF < 30^: The Hb levels in APW with ID (PF < 30 ng/mL). Hb^PF≥30^: the Hb levels in APW without ID (PF ≥ 30 ng/mL). PF^*^: the PF levels in APW without ID (PF ≥ 30 ng/mL). PF: the PF levels in all APW. –α^3.7(or4.2)^/αα, –α^3.7^/αα and –α^4.2^/αα were combined into one group. α^CS(orQS)^α/αα, α^CS^α/αα and α^QS^α/αα were combined into one group. –α^3.7(or4.2)^/–α^3.7(or4.2)^: –α^3.7^/–α^3.7^, –α^3.7^/–α^4.2^, and –α^4.2^/–α^4.2^ were combined into one group. α^CS(orQS)^α/–α^3.7(or4.2)^: α^CS^α/–α^3.7^, α^CS^α/–α^4.2^, α^QS^α/–α^3.7^, and α^QS^α/–α^4.2^ were combined into one group. α^WS^α/–α^3.7(or4.2)^: α^WS^α/–α^3.7^, and α^WS^α/–α^4.2^ were combined into one group. –α^3.7(or4.2)^/–^SEA^: –α^3.7^/–^SEA^, and -α^4.2^/–^SEA^ were combined into one group. β^T4^/β: β^IVS − II − 654(C>T)^/β, β^CD41/42(−TTCT)^/β, β^CD17(A>T)^/β, and β^CD71/72(+A)^/β were combined into one group. ^a^*P* < 0.05 vs. the group of −α^3.7(or4.2)^/–^SEA^. ^b^*P* < 0.05 vs. the Normal group. ^c^*P* < 0.05 vs. the groups of β^T4^/β and β^−28(A>G)^/β. ^d^*P* < 0.05 vs. the group of β^T4^/β. /: statistical analysis was performed when the sample size was greater than 5. Hb, hemoglobin; PF, plasma ferritin; APW, anemic pregnant women; ID, iron deficiency.

In the published article, there was an error. A correction has been made to the section Results, subsection “Analysis of anemia prevalence and hematological phenotype indexes in pregnant women with various thalassemia genotypes”, paragraph 1. This sentence previously stated:

“The anemia rates of αα/–^SEA^, –α^3.7^/–α^3.7^, –α^3.7^/–α^4.2^, and –α^4.2^/–α^4.2^ ranged from 35.96 to 37.82% (*P* < 0.05).”

The corrected sentence appears below:

“The anemia rates of αα/–^SEA^, –α^3.7^/–α^3.7^, –α^3.7^/–α^4.2^, and –α^4.2^/–α^4.2^ ranged from 35.96 to 37.82% (*P* > 0.05).”

A correction has been made to the section Discussion, subsection “Analysis of anemia prevalence and hematological phenotype indexes of pregnant women with various thalassemia genotypes”, paragraph 1. This sentence previously stated:

“The anemia rate among carriers of αα/–^SEA^, –α^3.7^/–α^3.7^, –α^4.2^/–α^4.2^, and –α^3.7^/–α^4.2^ ranged from 35.96 to 37.82% (*P* < 0.05), and no significant differences were observed in Hb levels among those genotypes.”

The corrected sentence appears below:

“The anemia rate among carriers of αα/–^SEA^, –α^3.7^/–α^3.7^, –α^4.2^/–α^4.2^, and –α^3.7^/–α^4.2^ ranged from 35.96 to 37.82% (*P* > 0.05), and no significant differences were observed in Hb levels among those genotypes.”

A correction has been made to the section Discussion, subsection “Prevalence of iron deficiency in anemic pregnant women with various genotypes of thalassemia”, paragraph 1. This sentence previously stated:

“Although there is no large sample, multicenter randomized controlled study that has yet confirmed the need for routine iron supplementation in PW with thalassemia minor genotypes, we suggest that the iron load of APW with β- thalassemia minor genotypes should be monitored dynamically during iron supplementation”

The corrected sentence appears below:

“Although there is no large sample, multicenter randomized controlled study that has yet confirmed the need for routine iron supplementation in PW with thalassemia minor genotypes, we suggest that the ferritin levels of APW with β- thalassemia minor genotypes should be monitored dynamically during iron supplementation”

The authors apologize for these errors and state that this does not change the scientific conclusions of the article in any way. The original article has been updated.

