# Supplementary figures and images for: Prevalence of iron-deficiency anemia in pregnant women with various thalassemia genotypes: Thoughts on iron supplementation in pregnant women with thalassemia genes
Source: Front Nutr. 2022 Nov 17;9:1005951. doi: 10.3389/fnut.2022.1005951 (PMC9713633; doi:10.3389/fnut.2022.1005951)

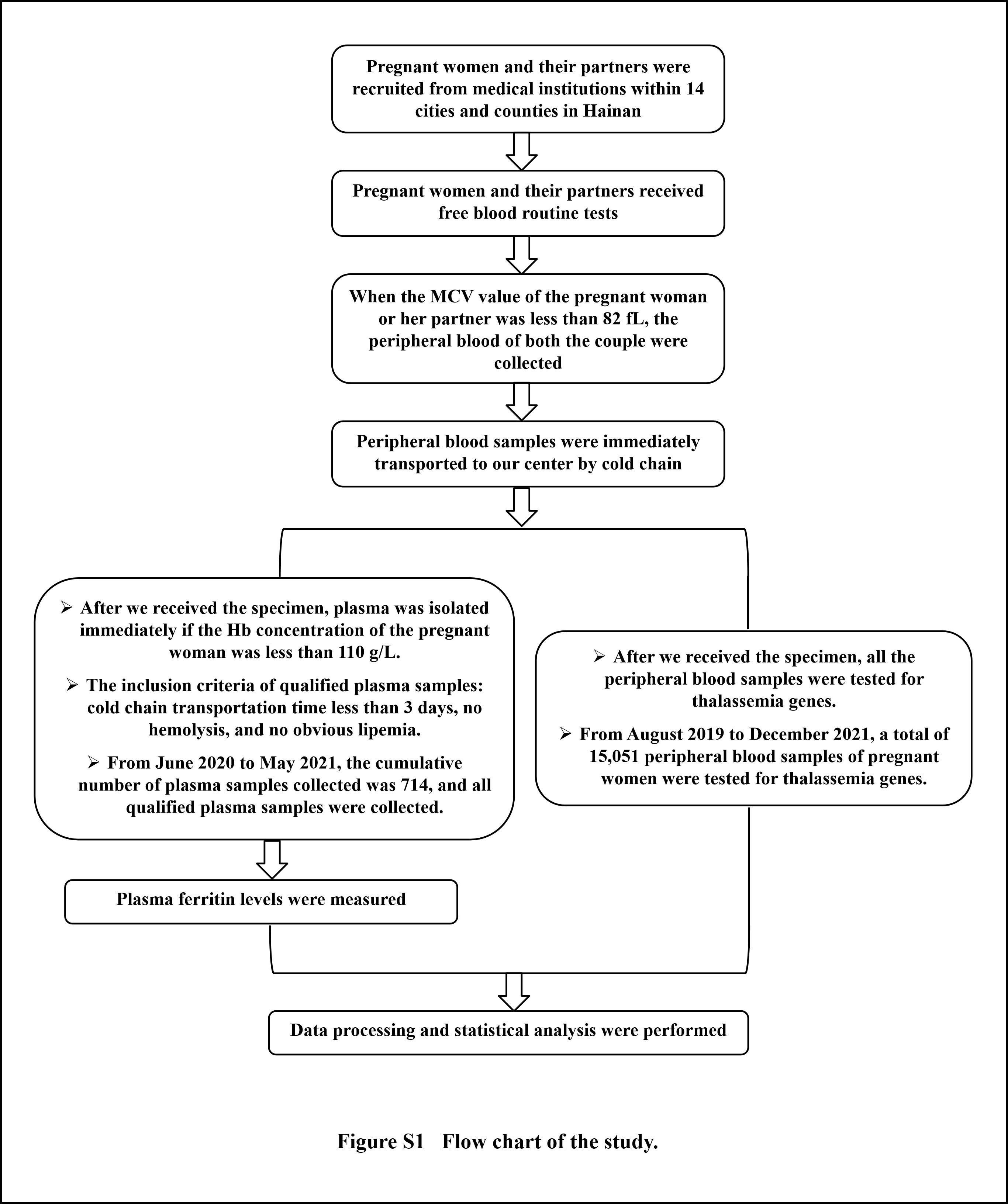

Supplement: Supplementary file 1 [file Image_1.JPEG]
